# Estimation of intrinsic water-use efficiency from δ^13^C signature of C_3_ leaves: Assumptions and uncertainty

**DOI:** 10.3389/fpls.2022.1037972

**Published:** 2023-01-12

**Authors:** Wei Ting Ma, Yong Zhi Yu, Xuming Wang, Xiao Ying Gong

**Affiliations:** ^1^ Key Laboratory for Subtropical Mountain Ecology (Ministry of Science and Technology and Fujian Province Funded), College of Geographical Sciences, Fujian Normal University, Fuzhou, China; ^2^ Key Laboratory for Humid Subtropical Eco-Geographical Processes of the Ministry of Education, Fujian Normal University, Fuzhou, China; ^3^ Fujian Provincial Key Laboratory for Plant Eco-physiology, Fuzhou, China

**Keywords:** water-use efficiency, carbon isotope discrimination, mesophyll conductance, post-photosynthetic fractionation, climate change, photosynthesis

## Abstract

Carbon isotope composition (δ^13^C) has been widely used to estimate the intrinsic water-use efficiency (iWUE) of plants in ecosystems around the world, providing an ultimate record of the functional response of plants to climate change. This approach relies on established relationships between leaf gas exchange and isotopic discrimination, which are reflected in different formulations of ^13^C-based iWUE models. In the current literature, most studies have utilized the simple, linear equation of photosynthetic discrimination to estimate iWUE. However, recent studies demonstrated that using this linear model for quantitative studies of iWUE could be problematic. Despite these advances, there is a scarcity of review papers that have comprehensively reviewed the theoretical basis, assumptions, and uncertainty of ^13^C-based iWUE models. Here, we 1) present the theoretical basis of ^13^C-based iWUE models: the classical model (iWUE_sim_), the comprehensive model (iWUE_com_), and the model incorporating mesophyll conductance (iWUE_mes_); 2) discuss the limitations of the widely used iWUE_sim_ model; 3) and make suggestions on the application of the iWUE_mes_ model. Finally, we suggest that a mechanistic understanding of mesophyll conductance associated effects and post-photosynthetic fractionation are the bottlenecks for improving the ^13^C-based estimation of iWUE.

## Introduction

During photosynthesis, plant stomata act as a control valve for the diffusion of CO_2_ and water vapor, regulating the rates of water and carbon exchange between the biosphere and the atmosphere (de Boer et al., 2011; Adams et al., 2020; Walker et al., 2021). Intrinsic water-use efficiency (iWUE), defined as the ratio of net photosynthetic rate (*A*_n_) to stomatal conductance for water vapor (*g*_sw_), plays a key role in quantifying carbon uptake and water loss at leaf to continental scales (Seibt et al., 2008; Keenan et al., 2013). The response of iWUE is fundamental to climate change research since small changes in iWUE can have profound impacts on global carbon and water cycles. Furthermore, iWUE can provide insights into the mechanisms of plant physiological responses to climate change and support the screening and breeding of climate-resilient crops (Farquhar and Richards, 1984; von Caemmerer et al., 2014; Gresset et al., 2014). Central to these research domains is the quantification of iWUE.

Stable carbon isotope discrimination (Δ) can be used as an integrated measure of iWUE in C_3_ plants (Farquhar et al., 1989). Plants discriminate against ^13^C in favour of ^12^C during photosynthetic CO_2_ assimilation in C_3_ leaves, and the variation in carbon isotope composition (δ^13^C) from source CO_2_ to photosynthetic products (e.g., bulk leaf organic carbon or sugars) is termed as Δ, following Farquhar et al. (1982b); Farquhar et al. (1989):


Equation 1 $$\Delta =\frac{\delta^{13}C_{\text{a}}-\delta^{13}C_{\text{p}}}{1+\delta^{13}C_{\text{p}}}$$

where atmospheric δ^13^C_a_ is approximately -7~-8‰ during the 20th century. Δ can also be estimated from δ^13^C of CO_2_ entering (δ_in_ and C_in_) and leaving (δ_out_ and C_out_) the cuvette during gas exchange, termed as online ^12^C/^13^C discrimination (Evans et al., 1986):


Equation 2 $$\Delta_{\text{online}}=\frac{\xi \left(\delta_{\text{out}}-\delta_{\text{in}}\right)}{1+\delta_{\text{out}}-\;\xi \left(\delta_{\text{out}}-\delta_{\text{in}}\right)}$$

where *ξ*= *C*_in_/(*C*_in_-*C*_out_). In this way, Δ can be measured nondestructively to probe real-time responses of photosynthesis at high temporal resolution. Changes in photosynthetic parameters (*A*_n_ and *g*_s_) are captured in Δ_online_ and the isotopic signatures are further imprinted on plant tissues during biosynthesis. As such, biomass-based Δ reflects physiological status of plants throughout the growth period of plant tissues (Cernusak et al., 2013; Soh et al., 2019). Different from classical approaches such as gas exchange or growth analysis, biomass-based Δ can be applied retrospectively, providing a useful record of iWUE at large spatial and temporal scales (Frank et al., 2015; Adams et al., 2020; Gong et al., 2022).

Inferring iWUE from isotopic records relies on theoretical models. In the current literature, most studies have utilized the simple, linear equation of photosynthetic discrimination to estimate iWUE. However, it can be problematic to interpret iWUE using this linear model which ignores effects other than diffusion through stomata and carboxylation. For instance, Seibt et al. (2008) suggested that the uncertainty in iWUE-^13^C models was related to the simplification of mesophyll conductance (*g*_m_). *g*_m_ represents the conductance to CO_2_ diffusion from the intercellular space to the carboxylation site in chloroplasts, a key limiting factor of photosynthesis in addition to stomatal conductance and biochemical capacity (Tholen et al., 2012; Stangl et al., 2019). However, recent advances in δ^13^C-based iWUE estimation have not been systematically reviewed. The main objective of this mini review is to concisely summarize the theoretical basis and uncertainties of δ^13^C-based iWUE models. We (i) present different formulations of Δ and the associated assumptions, (ii) present Δ-based iWUE models derived from those formulations: the classical model (iWUE_sim_), the comprehensive model (iWUE_com_), the model incorporating *g*_m_ (iWUE_mes_), (iii) discuss the limitations of the widely used iWUE_sim_ model; and make suggestions on the application of the iWUE_mes_ model.

## Comprehensive model of photosynthetic ^13^C discrimination and simplifications

A comprehensive description of ^13^C discrimination (Δ_com_) during C_3_ photosynthesis was given by Farquhar et al. (1982b) and extended to include ternary effects of transpiration on CO_2_ assimilation by Farquhar and Cernusak (2012):


Equation 3 $$\Delta_{\text{com}}=\frac{1}{1-t}\left(a_{ac}\frac{C_{a}-C_{i}}{C_{a}}\right)+\frac{1+t}{1-t}\left(a_{m}\frac{C_{i}-C_{c}}{C_{a}}+b\frac{C_{c}}{C_{a}}-\frac{\alpha_{b}}{\alpha_{e}}e\frac{R_{d}}{V_{c}}\frac{C_{c}}{C_{a}}-\frac{\alpha_{b}}{\alpha_{f}}f\frac{{\text{Г}}^{*}}{C_{a}}\right)$$

and


Equation 4 $$t=\frac{\left(1+a_{ac}\right)E}{2g_{ac}}$$

**Figure 1:**
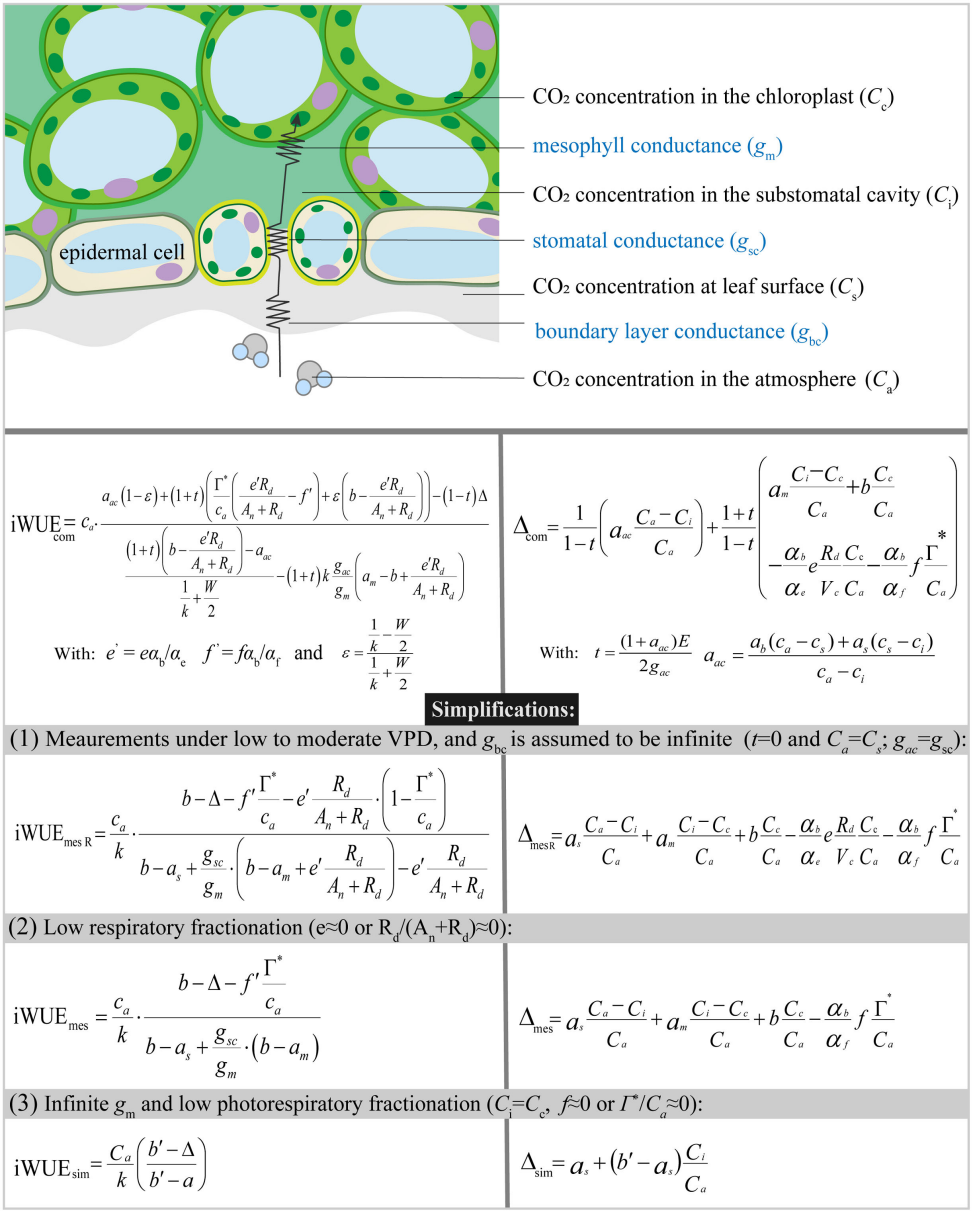
Diagram of the CO_2_ diffusion pathway in C_3_ leaves and different formulations of iWUE (iWUE_com_, iWUE_mes R_, iWUE_mes_, and iWUE_sim_) derived from the Farquhar et al. model for photosynthetic ^13^C discrimination.


Equation 5 $$a_{ac}=\frac{a_{b}\left(C_{a}-C_{s}\right)+a_{s}\left(C_{s}-C_{i}\right)}{C_{a}-C_{i}}$$

where *a*_b_ (2.9‰) and *a*_s_ (4.4‰) are fractionations associated with the diffusion of CO_2_ through leaf boundary layer and in the air, respectively. *a*_m_ (1.8‰) is the fractionation associated with the dissolution and diffusion of CO_2_ in mesophyll (see **Table S1** for the list of parameters). *C*_a_, *C*_s_, *C*_i_ and *C*_c_ represent the mole fraction of CO_2_ in air, at leaf surface, in the intercellular spaces and chloroplast, respectively (**Figure 1**). Δ_com_ can be separated into a series of fractionation components of leaf boundary layer conductance (Δ*_g_*_bc_), stomatal conductance (Δ*_g_*_sc_), mesophyll conductance (Δ*_g_*_m_), Rubisco (ribulose-1,5-bisphosphate carboxylase/oxygenase) carboxylation (Δ*_b_*), day respiration (Δ*_e_*), and photorespiration (Δ*_f_*). Note that *t* is included to account for the ternary effects of transpiration rate (*E*) on photosynthetic discrimination (Farquhar and Cernusak, 2012). Usually, the effect of *t* is small and can be omitted under low or moderate vapor pressure deficit (VPD) (Farquhar and Cernusak, 2012; Evans and Caemmerer, 2013). If Δ*_g_*_bc_ is also omitted (*C*_a_=*C*_s_ and *g*_ac_=*g*_sc_), the Δ_com_ model is simplified as:

Equation 6 $$\Delta_{\text{mes R}}=a_{s}\frac{C_{a}-C_{i}}{C_{a}}+a_{m}\frac{C_{i}-C_{c}}{C_{a}}+b\frac{C_{c}}{C_{a}}-\frac{\alpha_{b}}{\alpha_{e}}e\frac{R_{d}}{V_{c}}\frac{C_{c}}{C_{a}}-\frac{\alpha_{b}}{\alpha_{f}}f\frac{{\text{Г}}^{*}}{C_{a}}$$

where the subscript “mes R” indicates that the expression takes mesophyll conductance, day respiration and photorespiration into account.

Δ_e_, the respiratory contribution to discrimination is mainly determined by respiratory fractionation (*e*), and *R*_d_/(An+ *R*_d_). Δ_e_ has rarely been accurately quantified largely due to the difficulty of estimating *R*_d_ (Tcherkez et al., 2017; Gong et al., 2018). Moreover, fractionation of day respiration has rarely been reported, and *e* estimated from respiration in the dark varies between 0 and -6‰ (Ghashghaie et al., 2003; Tcherkez et al., 2010). Under natural conditions, Δ_e_ is usually small and negligible (Seibt et al., 2008; Ubierna and Farquhar, 2014). Notably, a significant apparent respiratory fractionation may occur when the CO_2_ source used for combined gas exchange and isotopic measurements has a δ^13^C differed from that of the ambient air (Gillon and Griffiths, 1997; Gong et al., 2015). Under such conditions, *e* should be corrected to account for the isotopic disequilibria between photosynthetic and respiratory fluxes (Wingate et al., 2007; Gong et al., 2015). Assuming Δ_e_=0, Equation 6 can be simplified as:


Equation 7 $$\Delta_{\text{mes}}=a_{s}\frac{C_{a}-C_{i}}{C_{a}}+a_{m}\frac{C_{i}-C_{c}}{C_{a}}+b\frac{C_{c}}{C_{a}}-\frac{\alpha_{b}}{\alpha_{f}}f\frac{{\text{Г}}^{*}}{C_{a}}$$

*C*_c_ is usually unknown since its calculation requires *g*_m_ which cannot be directly measured. *g*_m_ is assumed to be infinite in early studies (for a review see Flexas et al., 2012); that is, CO_2_ mole fraction in the chloroplast is equal to that in the intercellular space. Assuming *C*_i_=*C*_c_ and Δ*_f_* =0, Equation 7 is simplified as:


Equation 8 $$\Delta_{\text{sim}}=a_{s}+\left(b'-a_{s}\right)\frac{C_{i}}{C_{a}}$$

## Comprehensive model of iWUE and simplifications

The comprehensive model of iWUE which includes all fractionation components of Equation 3 was first derived by Ma et al. (2021):


Equation 9 $${\text{iWUE}}_{\text{com}}=c_{a}\frac{a_{ac}(1-є)+(1+t)\left[\frac{{Г}^{*}}{c_{a}}\left(\frac{e'R_{d}}{A_{n}+R_{d}}-f'\right)+є\left(b-\frac{e'R_{d}}{A_{n}+R_{d}}\right)\right]-(1-t)\Delta }{\frac{(1+t)\left(b-\frac{e'R_{d}}{A_{n}+R_{d}}\right)-a_{ac}}{\frac{1}{k}+\frac{W}{2}}-(1+t)k\frac{g_{ac}}{g_{m}}\left(a_{m}-b+\frac{e'R_{d}}{A_{n}+R_{d}}\right)}$$

where *e*’=*ea_b_*/*a*_e_, *f*’=*fa_b_*/*a_f_* and є=(1/k-*W*/2)/(1/k+*W*/2). This formulation is particularly useful for assessing the contribution of each fractionation component to iWUE estimates. Ma et al. (2021) performed sensitivity tests using theoretical data of the standard photosynthetic scenarios. Their results indicated that ternary correction and Δ*_g_*_bc_ had little influence on iWUE_com_ estimates (error< 2 μmol mol^-1^), which is in agreement with Seibt et al. (2008). Neglecting the contribution of *t* and Δ*_g_*_bc_, Equation 9 can be simplified as:


Equation 10 $${\text{ iWUE}}_{\text{mes R}}=\frac{c_{a}}{k}\cdot \frac{b-\Delta -f'\frac{\Gamma^{*}}{c_{a}}-e'\frac{R_{d}}{A_{n}+R_{d}}\left(1-\frac{\Gamma^{*}}{c_{a}}\right)}{b-a_{s}+\frac{g_{sc}}{g_{m}}\left(b-a_{m}+e'\frac{R_{d}}{A_{n}+R_{d}}\right)-e'\frac{R_{d}}{A_{n}+R_{d}}}$$

Δ_e_ in the iWUE_mes R_ model could be ignored as it caused an error of less than 2 μmol mol^-1^ in typical photosynthetic scenarios (Ma et al., 2021). Excluding the contribution of day respiratory, Equation 10 can be simplified as:


Equation 11 $${\text{iWUE}}_{\text{mes }}=\frac{c_{a}}{k}\cdot \frac{b-\Delta -f'\frac{\Gamma^{*}}{c_{a}}}{b-a_{s}+\frac{g_{sc}}{g_{m}}\left(b-a_{m}\right)}$$

The iWUE_mes_ model provided iWUE estimates that are numerically very similar with iWUE_com_ (error< 3 μmol mol^-1^) (Ma et al., 2021). Neglecting the contribution of *g*_m_ and photorespiration, the simplified equation for iWUE is given as:


Equation 12 $${\text{iWUE}}_{\text{sim}}=\frac{C_{\text{a}}}{k}\left(\frac{b'-\Delta }{b'-a}\right)$$

This linear relationship between iWUE and photosynthetic ^13^C discrimination is the most used to estimate iWUE, however, the limitations of this formulation have been raised (Seibt et al., 2008; Ubierna and Farquhar, 2014; Ma et al., 2021).

## Uncertainty in iWUE_sim_ estimation associated with mesophyll conductance

Experimental evidence shows that *g*_m_ exerts a significant limitation on CO_2_ diffusion and leads to a significant drawdown from *C*_i_ to *C*_c_ (Loreto et al., 1992; Flexas et al., 2008; Cano et al., 2014). It is apparent from Equation 11 that, assuming an infinite *g*_m_ will lead to overestimation of iWUE, this is supported by experimental observations (Barbour et al., 2010; Stangl et al., 2019; Adams et al., 2020). Experimental results showed that the relationship between Δ and water-use efficiency is at least partly a function of *g*_m_ (Warren and Adams, 2006). Therefore, it is important to incorporate *g*_m_ in the parameterization of the iWUE model. Ma et al. (2021) showed that iWUE_sim_ overestimated iWUE by c. 65%. Importantly, the magnitude of overestimation is dependent on Δ, making correction using empirical relations difficult. These results raise concerns regarding the accuracy of iWUE_sim_ estimations.

The overestimation of the iWUE_sim_ model has also been observed in recent studies using ^13^C series of environmental archives (Baca Cabrera et al., 2021; Bing et al., 2022). More importantly, iWUE_sim_ model could provide biased estimations of historical iWUE trend. Gong et al. (2022) analyzed tree ring ^13^C series across the globe using the iWUE_mes_ model, and reported that iWUE_sim_ model significantly overestimated iWUE (by c. 100%) and the rate of iWUE gain with time or *C*_a_ (by c. 70%) during the 20th century. This finding has been confirmed by studies carried out in distinct ecosystems (Bing et al., 2022; Mathias and Hudiburg, 2022). Failure to consider *g*_m_ must lead to an overestimated historical trend as implied by the partial derivative of iWUE_mes_ (Equation 11) (Gong et al., 2022):


Equation 13 $$\frac{d\text{iWUE}}{dC_{a}}=\frac{b-\Delta }{k\left(\frac{g_{sc}}{g_{m}}\left(b-a_{m}\right)+b-a_{s}\right)}$$

Given that ^13^C series of environmental archives (e.g. tree rings) provide a unique proxy for benchmarking the output of land surface models (Frank et al., 2015; Wang et al., 2017; Lavergne et al., 2022), cautions should be paid when iWUE_sim_ model is used to predict historical trend of iWUE.

## Uncertainty in iWUE_sim_ estimation associated with *b’*

The value and physiological meaning of *b’* in the equation of Δ_sim_ or iWUE_sim_ remain subjects of debate. Initially, Farquhar et al. (1982a) proposed that *b’* (27‰) could be derived from early *in vitro* estimations of Rubisco carboxylation. As it agreed well with the relationship between measured biomass-based Δ and *C*_i_/C_a_, it was interpreted as a fitted value. However, when measured Δ from online instantaneous measurements was used to fit Equation 8, the fitted *b’* appears to be lower than 27‰ (Caemmerer and Evans, 1991; Ma et al., 2021).

*b’* was also explained as the net fractionation caused by Rubisco and PEPC (phosphoenolpyruvate carboxylase). Farquhar and Richards (1984) described *b’* as a function of relative contribution of Rubisco and PEPC carboxylation:


Equation 14 $$b'=\left(1-\beta \right)b+\beta b_{4}$$

where *b* (29-30‰) and *b*_4_ (usually taken as -5.7‰ at 25°C) are fractionation factors of Rubisco and PEPC carboxylation, respectively. *β* is the proportion of carbon fixation through PEPC carboxylation. PEPC uses $\text{HC}{\text{O}}_{3}^{-}$ produced by CO_2_ hydration as the substrate for the synthesis of aspartate or malate, which is important for the control of cellular pH (Davies, 1979). Generally, carboxylation by Rubisco contributes a greater fraction of carbon in plants and respiratory substrates. But it is also suggested that the PEPC carboxylation could be important under the conditions of low stomatal conductance or carboxylation in darkness (Ikeda and Yamada, 1981; Gupta et al., 1994; Hibberd and Quick, 2002). Furthermore, several studies have revealed that N source and concentration were potential factors affecting carbon fixation by PEPC, which indicates that *b’* could vary with nitrogen metabolism (Raven and Farquhar, 1990; Douthe et al., 2012; Lian et al., 2021). That is, Equation 14 is not particularly useful for iWUE estimation because *β* is variable and difficult to quantify.

*b’* has also been described by Ubierna and Farquhar (2014) as a parameter that included carboxylation, mesophyll conductance, and photorespiration:


Equation 15 $$b'≅b\frac{C_{\text{c}}}{C_{\text{i}}}+a_{\text{m}}\left(1-\frac{C_{\text{c}}}{C_{\text{i}}}\right)-f\frac{{\text{Г}}^{*}}{C_{a}}$$

According to Equation 15, *b’* is largely dependent on *C*_c_/*C*_i_ which is modulated by *g*_m_. It should be noted that the most used *b*’=27‰ is consistent with the *C*_c_/C_i_ value of 0.9, higher than the common values of 0.7-0.8 (Caemmerer and Evans, 1991; Warren et al., 2003). So far, Equations 14 and 15 have only been used to discuss the potential origin of variation in *b*’, but have not been incorporated in the model of iWUE estimation. In short, there is still no consensus concerning the interpretation of *b*’, and current discussion on *b’* (Equations 14, 15) illustrated that it should not be treated as a constant value of 27‰.

## Uncertainty in iWUE associated with post-photosynthetic fractionation

Post-photosynthetic fractionation (Δ_post_) includes the discrimination processes that follow photosynthetic carbon fixation, altering δ^13^C signals in plant organs and leaves at different development stages (Badeck et al., 2005; Vogado et al., 2020). In general, heterotrophic organs (branches, stems and roots) are ^13^C-enriched compared with autotrophic organs (leaves) (Badeck et al., 2005; Bowling et al., 2008; Cernusak et al., 2009; Lamade et al., 2016), and the immature leaves (heterotrophic phase) are ^13^C-enriched (by *c.* 2‰) compared to mature leaves (autotrophic phase) in both deciduous and evergreen species (Lamade et al., 2009; Vogado et al., 2020). However, the contribution of Δ_post_ to δ^13^C of plant tissues and its influence on iWUE estimation are poorly understood.

Several studies has accounted for Δ_post_ to estimate iWUE (**Table S2**). Gimeno et al. (2021) found an improvement in correlation between iWUE estimated from gas exchange and that from Δ when *g*_m_ and Δ_post_ were accounted for. In that study, Δ_post_ was taken as -2.5‰ estimated from the δ^13^C difference between phloem contents and whole-tree photosynthesis. Similarly, δ^13^C of wood and cellulose have been corrected by -3.2‰ and -1.3‰, respectively, to account for the offset from leaf δ^13^C (Thomas et al., 2013; Brownlee et al., 2016). In other studies, iWUE was calculated from tree-ring with a δ^13^C offset of -2‰ to account for Δ_post_ (Michelot et al., 2011; Frank et al., 2015). Without correcting a Δ_post_ of about 2.5‰, iWUE estimated from the δ^13^C of tree-ring could be overestimated by 20% (Gessler et al., 2009).

The likely mechanisms underlying Δ_post_ include fractionation associated with respiration, transport and mixing of assimilates (Tcherkez et al., 2003; Brüggemann et al., 2011; Bögelein et al., 2019). Respiratory fractionation ranges from -6 to 0‰ in various species (Duranceau et al., 1999; Ghashghaie et al., 2001; Tcherkez et al., 2003). Also, there are some variations in the apparent fractionation during transport processes (e.g., day-night differences in δ^13^C of leaf-export organic matter and different leaf-to-phloem δ^13^C signatures along vertical canopy gradients) and very few direct measurements of isotopic differences between components at molecule/atom scale (Gessler et al., 2008; Mauve et al., 2009; Gilbert et al., 2011; Gilbert et al., 2012; Bögelein et al., 2019). In addition, post-photosynthetic fractionation is complicated by ontogenic effects (e.g., size, height, and age of individuals) that can confound the relationship between iWUE and environmental factors (Vadeboncoeur et al., 2020). That is, the influence of Δ_post_ could accumulate over time and lead to age-dependent patterns (Cernusak et al., 2009). Therefore, using a constant, empirical value of Δ_post_ could be unreliable. A mechanistic description of Δ_post_ should be very useful to improve the iWUE estimates, which requires further study on the fractionations associated with respiration, transport and allocation of assimilates.

## iWUE_mes_, a useful, simplified model for estimating iWUE

iWUE_mes_ takes the influence of mesophyll conductance and photorespiratory fractionation into account. We propose to use the iWUE_mes_ model (Equation 11) since it considers the components that have a significant influence on the iWUE estimation. In particular, it includes *g*_m_ effect which is known to affect iWUE prediction.

Parameterizing the iWUE_mes_ model requires *g*_sc_/*g*_m_ rather than *g*_m_ (Ma et al., 2021). Some studies use a constant *g*_m_ in the equation to estimate iWUE (Keeling et al., 2017), which makes more sense than disregarding *g*_m_. However, the assumption of constant *g*_m_ is not supported by experimental evidence. The positive relationships between *g*_sc_ and *g*_m_ have been reported in many studies (Flexas et al., 2013; Gong et al., 2018), and this relationship is rather conserved across levels of CO_2_, irradiance, and drought stress and functionally distinct species (Flexas et al., 2008; Ma et al., 2021; Gong et al., 2022). Incorporating the *g*_sc_/*g*_m_ ratio improves the predictive accuracy of the iWUE model, as demonstrated in gas exchange experiments (Ma et al., 2021). Without knowing *g*_m_, it is preferable to use the average *g*_sc_/*g*_m_ of 0.79 ( ± 0.07) derived from a global synthesis to parameterize the iWUE_mes_ model rather than using the iWUE_sim_ model.

We acknowledge that using a constant *g*_sc_/*g*_m_ is not always adequate. As more *g*_m_ data become available, interspecific differences in *g*_sc_/*g*_m_ can be identified and should be accounted for in the estimated iWUE. Theoretically, species-specific *g*_sc_/*g*_m_ is more appropriate to be used in the iWUE_mes_ estimation. It is also noteworthy that short-term responses of *g*_m_ are still not well defined, implying that neglecting short-term variation in *g*_sc_/*g*_m_ might lead to errors in estimating iWUE at nonsteady-states, thus should be addressed in further work. Moreover, the iWUE_mes_ model does not account for the post-photosynthetic fractionation, due to a lack of knowledge on post-photosynthetic fractionation. Therefore, we recommend for biomass-based analyses to distinguish the age of organs to minimize the influence of post-photosynthetic fractionation.

## Conclusion remarks

The comprehensive model of photosynthetic ^13^C discrimination of Farquhar and Cernusak (2012) is a synthesis of current understanding, and provides the theoretical basis for estimating iWUE from the ^13^C composition of plant materials. The classical iWUE_sim_ model has been shown to strongly overestimate iWUE and its historical trends due to the neglect of *g*_m_ associated effect, limiting its use in quantitative studies. iWUE_mes_ is suggested as a useful, simplified model for quantitative estimation of iWUE, which has been included in a standardized, open-source tool (R package) for calculation of iWUE from stable isotope signatures (Mathias and Hudiburg, 2022). Nonetheless, the formulation of iWUE_mes_ could still be further improved. For example, a fixed, empirical *g*_sc_/*g*_m_ value could be replaced by species-specific values or mechanistic relationships derived from experimental results. ^13^C discrimination of plant material, combining with appropriate iWUE models, is also an ultimate tool for screening genetic resources to enhance the iWUE of crops under climate change scenarios. One of the primary questions is how *g*_sc_ and *g*_m_ of plants will respond to changes in temperature, water availability, and carbon dioxide concentration. Furthermore, the response of post-photosynthetic fractionation to climate change factors remains unknown. We conclude that mechanistic descriptions of *g*_m_ associated effect and post-photosynthetic fractionation are the bottlenecks for improving the ^13^C-based estimation of iWUE.

## Author contributions

XG conceptualized the topic of this review, WM and YY wrote the first draft, and all authors contributed to the writing and revision of the manuscript. All authors contributed to the article and approved the submitted version.
