# Supplementary material for: Estimation of intrinsic water-use efficiency from δ13C signature of C3 leaves: Assumptions and uncertainty
Source: Front Plant Sci. 2023 Jan 12;13:1037972. doi: 10.3389/fpls.2022.1037972 (PMC9877432; doi:10.3389/fpls.2022.1037972)
Supplement: Supplementary file 1 [file DataSheet_1.docx]

Table S1 List of parameters in the ^13^C discrimination and iWUE models

| Symbol | Definition | Units | Value |
| --- | --- | --- | --- |
| *C*_a_ | CO_2_ concentration in the atmosphere | µmol mol^-1^ | Variable |
| *C*_s_ | CO_2_ concentration at the leaf surface | µmol mol^-1^ | Variable |
| *C*_i_ | CO_2_ concentration in the intercellular spaces | µmol mol^-1^ | Variable |
| *C*_c_ | CO_2_ concentration in the chloroplast | µmol mol^-1^ | Variable |
| *a*_b_ | fractionation during CO_2_ diffusion across the boundary layer | ‰ | 2.9 |
| *a*_s_ | fractionation during CO_2_ diffusion through the stomata | ‰ | 4.4 |
| *a*_ac_ | combined fractionation for CO_2_ diffusion across the boundary layer and the stomata | ‰ | Variable |
| *a*_m_ | fractionation associated with CO_2_ dissolution and diffusion in the mesophyll | ‰ | 1.8 |
| *b* | fractionation by Rubisco carboxylation | ‰ | 29 |
| *e* | fractionation by mitochondrial respiration | ‰ | -6-0 |
| *f* | fractionation during photorespiration | ‰ | 11 |
| *α_b_* | *α_b_ =* 1+*b* | -- | 1.029 |
| *α_e_* | *α_e_ =* 1+*e* | -- | Variable |
| *α_f_* | *α_f_ =* 1+*f* | -- | 1.011 |
| *g*_m_ | mesophyll conductance | mol m^-2^ s^-1^ | Variable |
| *g*_bc_ | boundary layer conductance to CO_2_ | mol m^-2^ s^-1^ | Variable |
| *g*_sc_ | stomatal conductance to CO_2_ | mol m^-2^ s^-1^ | Variable |
| *g*_ac_ | conductance to CO_2_ of the stomata plus boundary layer | mol m^-2^ s^-1^ | Variable |
| *A*_n_ | net assimilation rate | µmol m^-2^ s^-1^ | Variable |
| *R*_d_ | day respiration rate | µmol m^-2^ s^-1^ | Variable |
| *Γ** | CO_2_ compensation point in the absence of mitochondrial respiration | µmol mol^-1^ | Variable |
| *t* | ternary correction factor | -- | Variable |
| *W* | leaf-to-air vapor concentration difference | mmol mol^-1^ | Variable |
| *E* | transpiration rate | mmol m^-2^ s^-1^ | Variable |
| *k* | ratio of diffusion coefficients for water vapour and CO_2_ in air | -- | 1.6 |

Table S2 Post-photosynthetic fractionation (Δ_post_) values used in iWUE estimation

| Species | Measured materials | Δ_post_ values (‰) | Differences in ^13^C-isotopic signature | References | Sources of Δ_post_ value |
| --- | --- | --- | --- | --- | --- |
| *Eucalyptus tereticornis* | phloem | -2.5±0.7 | photosynthesis - phloem | Gimeno *et al.*, (2021) | measured value |
| *Juniperus virginiana* | tree-ring | -3.2 | leaves - wood | Thomas *et al.*, (2013) | measured value |
| *Quercus petraea* | tree-ring | -1.91 | leaves - wood | Michelot *et al.*, (2011) | from Badeck *et al.*, (2005) |
| *Picea glauca* | cellulose | -1.33 | leaves - wood cellulose | Brownlee *et al.*, (2016) | measured value |
| *Quercus* and *Pinus* | tree-ring | -2.1±1.5 | leaves - wood cellulose | Frank *et al.*, (2015) | from a survey of literature and meta-data reviews |
| *Quercus petraea* | tree-ring | 1 | wood - lignin | Michelot *et al.*, (2011) | from Helle and Schleser (2004) |

Badeck, F.W., Tcherkez, G., Nogués, S., Piel, C., and Ghashghaie, J. (2005). Post-photosynthetic fractionation of stable carbon isotopes between plant organs—a widespread phenomenon. *Rapid Commun. Mass Spectrom* 19(11), 1381-1391. doi: 10.1002/rcm.1912.

Brownlee, A.H., Sullivan, P.F., Csank, A.Z., Sveinbjörnsson, B., and Ellison, S.B.Z. (2016). Drought-induced stomatal closure probably cannot explain divergent white spruce growth in the Brooks Range, Alaska, USA. *Ecology* 97(1), 145-159. doi: https://doi.org/10.1890/15-0338.1.

Frank, D.C., Poulter, B., Saurer, M., Esper, J., Huntingford, C., Helle, G., et al. (2015). Water-use efficiency and transpiration across European forests during the Anthropocene. *Nat. Clim. Change* 5(6), 579-583. doi: 10.1038/nclimate2614.

Gimeno, T.E., Campany, C.E., Drake, J.E., Barton, C.V.M., Tjoelker, M.G., Ubierna, N., et al. (2021). Whole-tree mesophyll conductance reconciles isotopic and gas-exchange estimates of water-use efficiency. *New Phytol* 229(5), 2535-2547. doi: https://doi.org/10.1111/nph.17088.

Helle, G., and Schleser, G. (2004). Beyond CO_2_-fixation by Rubisco - an interpretation of ^13^C/^12^C variations in tree rings from novel intra-seasonal studies on broad-leaf trees. *Plant Cell Environ*. 27, 367-380. doi: https://doi.org/10.1111/j.0016-8025.2003.01159.x

Michelot, A., Eglin, T., Dufrêne, E., Lelarge-Trouverie, C., and Damesin, C. (2011). Comparison of seasonal variations in water-use efficiency calculated from the carbon isotope composition of tree rings and flux data in a temperate forest. *Plant Cell Environ* 34(2), 230-244. doi: https://doi.org/10.1111/j.1365-3040.2010.02238.x.

Thomas, R. B., Spal, S. E., Smith, K. R., and Nippert, J. B. (2013). Evidence of recovery of *Juniperus virginiana* trees from sulfur pollution after the Clean Air Act. *Proc. Natl. Acad. Sci. U.S.A.* 110, 15319-15324. doi: 10.1073/pnas.1308115110.
